# Characterization of a Novel Esterase Rv0045c from *Mycobacterium tuberculosis*

**DOI:** 10.1371/journal.pone.0013143

**Published:** 2010-10-01

**Authors:** Jiubiao Guo, Xiangdong Zheng, Lipeng Xu, Zhongyuan Liu, Kehui Xu, Shentao Li, Tingyi Wen, Siguo Liu, Hai Pang

**Affiliations:** 1 School of Medicine, Tsinghua University, Beijing, China; 2 Harbin Veterinary Research Institute, Chinese Academy of Agriculture, Harbin, China; 3 Department of Immunology, School of Basic Medical Sciences, Capital Medical University, Beijing, China; 4 Department of Industrial Microbiology and Biotechnology, Institute of Microbiology, Chinese Academy of Sciences, Beijing, China; Monash University, Australia

## Abstract

**Background:**

It was proposed that there are at least 250 enzymes in *M. tuberculosis* involved in lipid metabolism. Rv0045c was predicted to be a hydrolase by amino acid sequence similarity, although its precise biochemical characterization and function remained to be defined.

**Methodology/Principal Findings:**

We expressed the Rv0045c protein to high levels in *E. coli* and purified the protein to high purity. We confirmed that the prepared protein was the Rv0045c protein by mass spectrometry analysis. Circular dichroism spectroscopy analysis showed that the protein possessed abundant β-sheet secondary structure, and confirmed that its conformation was stable in the range pH 6.0–10.0 and at temperatures ≤40°C. Enzyme activity analysis indicated that the Rv0045c protein could efficiently hydrolyze short chain *p*-nitrophenyl esters (C_2_–C_8_), and its suitable substrate was *p*-nitrophenyl caproate (C_6_) with optimal catalytic conditions of 39°C and pH 8.0.

**Conclusions/Significance:**

Our results demonstrated that the Rv0045c protein is a novel esterase. These experiments will be helpful in understanding ester/lipid metabolism related to *M. tuberculosis*.

## Introduction

*Mycobacterium tuberculosis* (*M. tuberculosis*), firstly discovered by Robert Koch [1], is a pathogenic species and the causative agent of most tuberculosis [2]. The World Health Organization (WHO) has recognized the global threat imposed by *M. tuberculosis*, and statistics show that about one-third of the world's population has been infected. It was reported by the WHO that the increasing rate of new clinical cases was 8 million each year, with at least 3 million people deaths [3], [4], [5]. *M. tuberculosis* has an unusual, waxy coating on the cell surface (primarily mycolic acid), which highlights that there must be a large number of enzymes involved in lipid metabolism. In 1998, the whole genome of *M. tuberculosis* H37Rv strain was sequenced by the Sanger Center and the Institut Pasteur, showing at least 250 enzymes related to lipid metabolism including extracellular secreted enzymes, integrated cell wall enzymes and intracellular esterases/lipases, compared with about 50 enzymes in *E. coli* [6], [7].

The genomic organization and gene functionality of *M. tuberculosis* are invaluable for understanding the slowly growing pathogen. Mycobacterial genes that are involved in lipid metabolism, cell division chromosomal partitioning, and secretion are more likely to be required for survival in mice [8], [9]. Lamichhane and colleagues detected 31 *M. tuberculosis* genes that were found to be required for *in vivo* survival in mouse lungs. Mutation of six of the Mycobacterial membrane protein (mmpL) family genes severely compromised the ability of the respective mutants to multiply in mouse lungs [9].

In 2007, a *M. tuberculosis* CDC1551 (or Rv2224c of H37Rv) gene, *MT2282*, was identified as a virulence gene belonging to the microbial esterase/lipase family with an active site consensus sequence of G-X-S-X-G. In fact, the esterase was a cell wall-associated carboxyl esterase rather than a protease as initially annotated. Further research found that the MT2282 esterase was required for bacterial survival in mice and full virulence of *M. tuberculosis* [10].

The Rv0045c protein is a putative hydrolase, probably involved in ester/lipid metabolism of *M. tuberculosis*. Alignment among amino acid sequences showed that the Rv0045c protein shares little amino acid sequence similarity with members of the esterase/lipase family identified in *Bacteria, Archaea, Eukaryotes* and some viruses [11], such as Aes acetyl-esterase from *E. coli* [12], and *mosquito* carboxylesterase Estα2^1^ (A_2_) [13]. Here, we experimentally characterized the Rv0045c protein via protein expression, purification, biochemical characterization and enzyme activity analysis, and finally demonstrated that Rv0045c is a novel esterase in *M. tuberculosis*.

## Results

### Expression and purification of the Rv0045c protein

**Figure 1:**
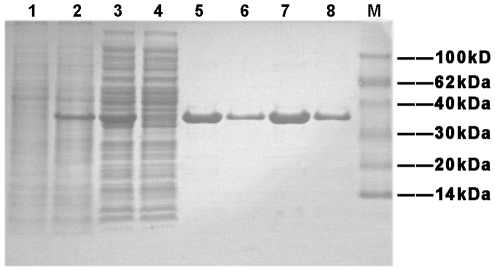
SDS-PAGE analysis for expression and affinity chromatography of the Rv0045c protein. Lane 1, culture pellet (uninduced); Lane 2, culture pellet (induced with 0.3 mM IPTG at 16°C); Lane 3, the supernatant of induced cells after sonication; Lane 4, fluid through Ni^2+^-affinity chromatography column; Lane 5 and 7: purified Rv0045c protein eluted by 20 mM Tris, 150 mM NaCl, 200 mM Imidazole, pH 7.5; Lane 6 and 8: purified Rv0045c protein eluted by 20 mM Tris, 150 mM NaCl, 500 mM Imidazole, pH 7.5; Lane M: molecular mass markers.

In order to allow easy purification and to attenuate the effect of a large tag on the biological activity of the Rv0045c protein, a 6×His-tag was chosen and added to its N-terminal. The fusion protein was overexpressed at 37°C and induced with 1 mM IPTG. SDS-PAGE analysis showed a major protein band with the expected 35.5 kDa size, but the recombinant Rv0045c protein was in form of inclusion bodies (data not shown). To make purification easy and to maintain the biological activity of the recombinant protein, the expression condition was optimized by raising the major fraction as a soluble protein under a feasible condition with 0.3 mM IPTG at 16°C (Fig. 1, lane 3).

**Figure 2:**
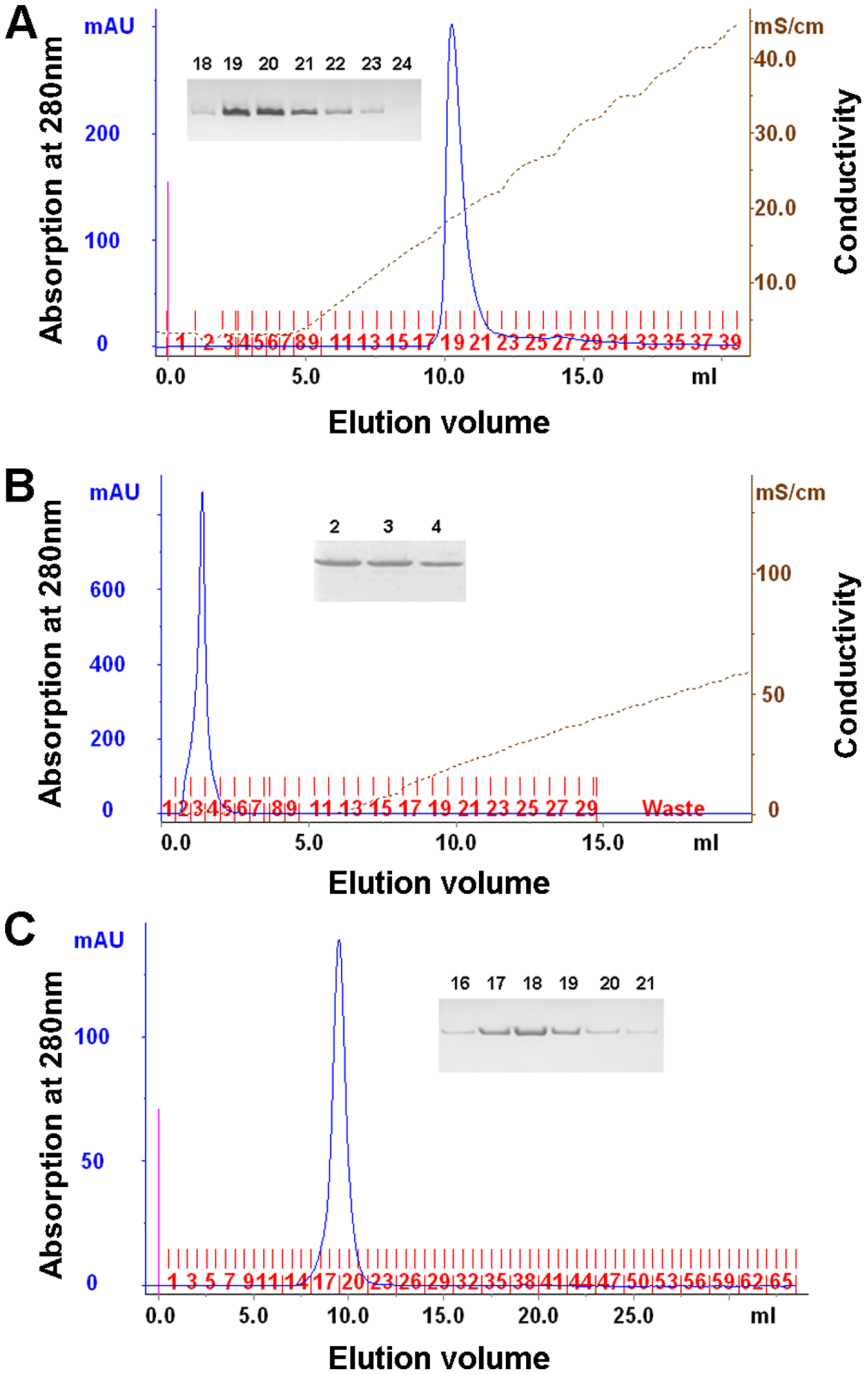
Purification of the Rv0045c protein by ion exchange chromatography and gel filtration chromatography. The Rv0045c protein was purified by anion exchange chromatography (A), cation exchange chromatography (B), and gel filtration chromatography (C). The purity was checked by SDS-PAGE analysis after each purification procedure.

First, we purified the soluble protein from supernatant using Ni^2+^-affinity chromatography (Fig. 1, lane 5 to lane 8). Subsequently, the eluted protein was concentrated, and loaded onto an anion exchange chromatography column (Fig. 2A) and a cation exchange chromatography column (Fig. 2B). Finally, the protein was further purified through gel filtration chromatography to >98% purity (Fig. 2C).

### MALDI-TOF mass spectrometry analysis of the Rv0045c protein

**Figure 3:**
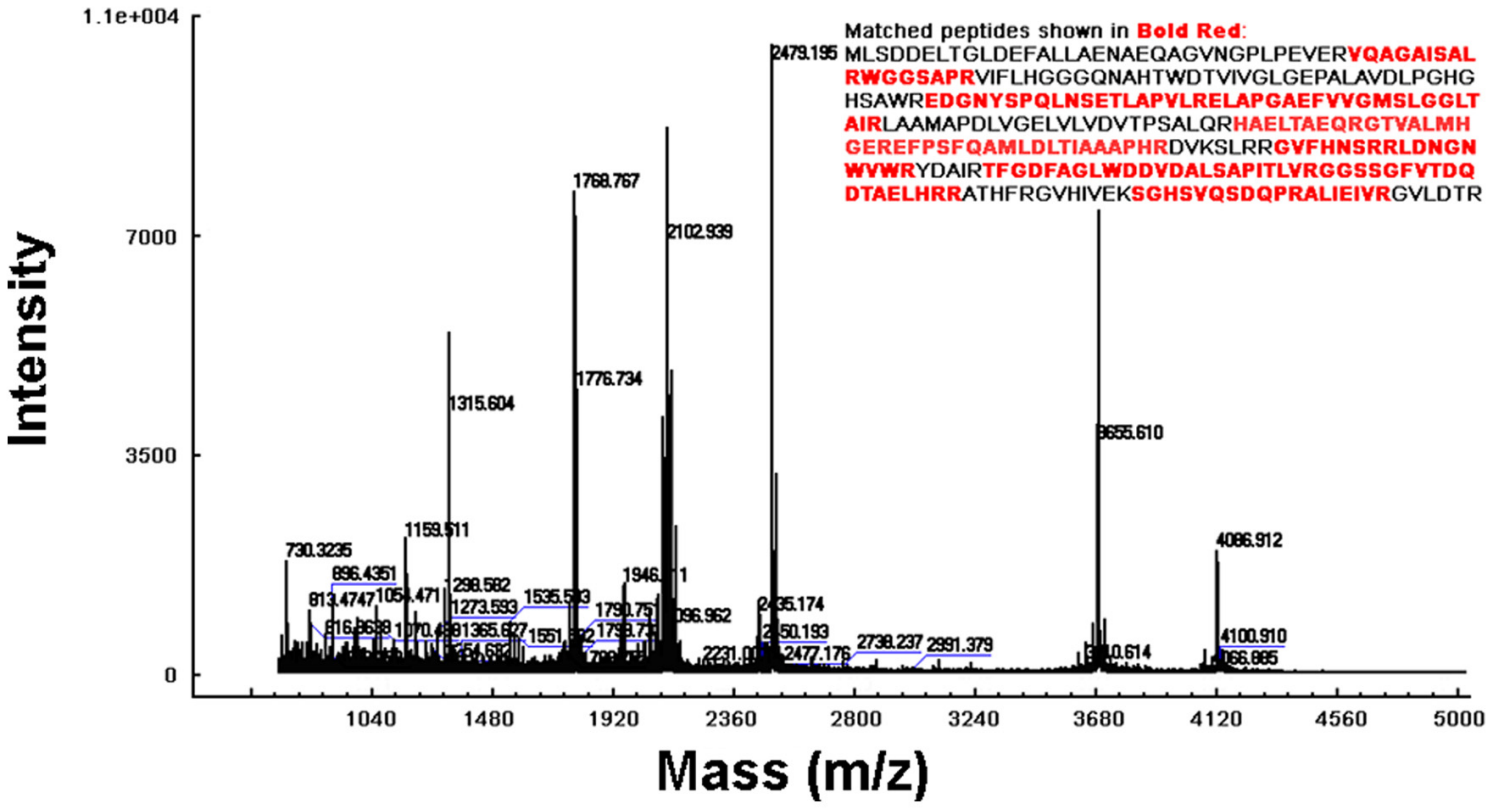
MALDI-TOF peptide mass fingerprint (PMF) spectrometry of the Rv0045c protein. The PMF analysis was made from fragments of purified Rv0045c protein derived through trypsin digestion. The expected tryptic masses clearly matched, with 1 Da tolerance, the calculated values. The sequence coverage of these fragments was shown in bold red.

We analyzed the purified Rv0045c protein by mass spectrometry. The MALDI-TOF MS spectrometry of the digested protein is shown in Fig. 3. The peptide mass fingerprinting (PMF) of the protein was observed and submitted to Mascot. Consequently, only NP_214559 protein from *M. tuberculosis* was obtained as a result with a score of 112. The results provided convincing evidence that the purified Rv0045c protein is the NP_214559 protein from *M. tuberculosis.*

### Circular dichroism spectroscopy analysis of the Rv0045c protein

**Figure 4:**
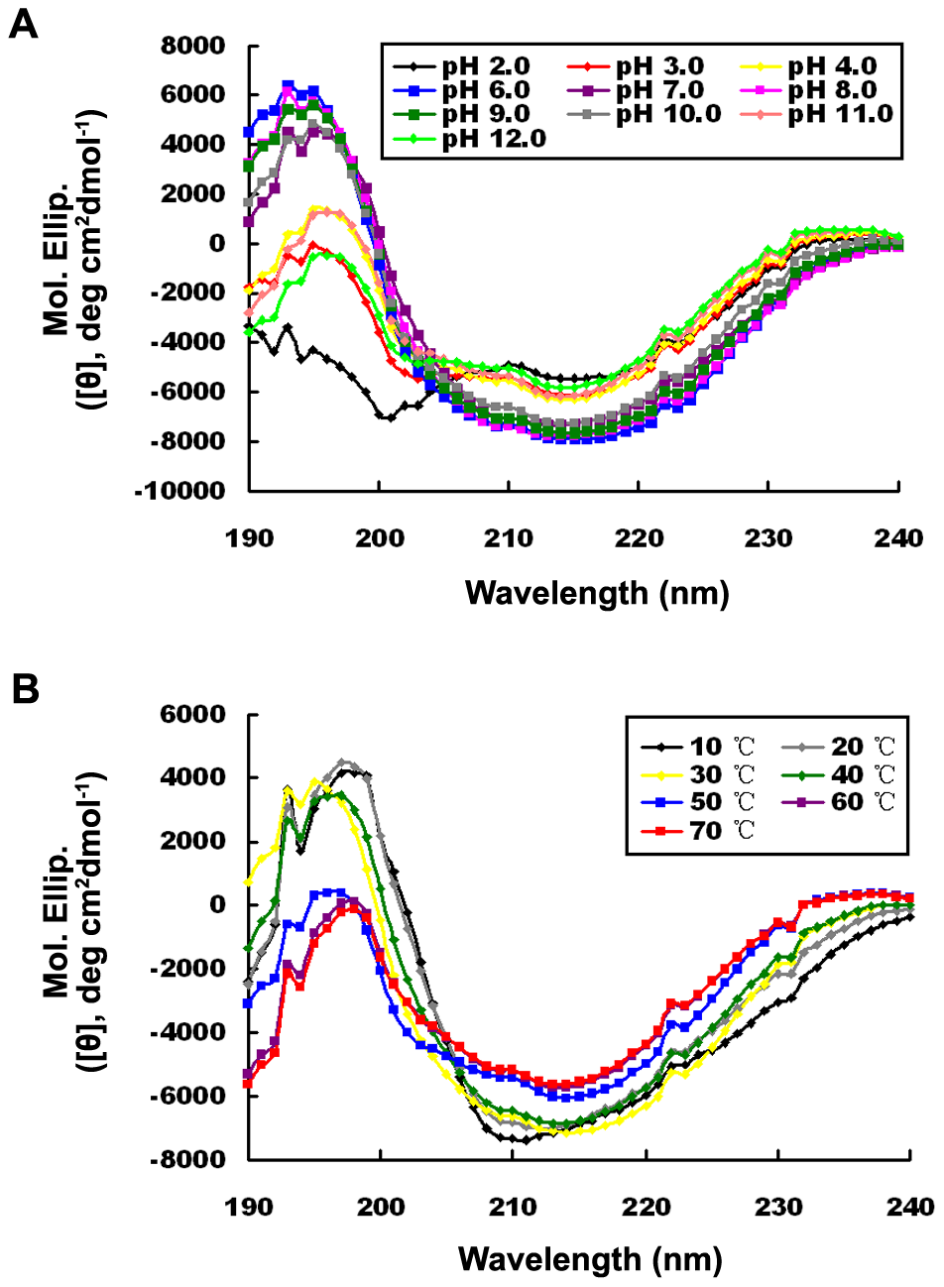
CD spectra of the Rv0045c protein at different pH and temperatures. The CD measurements were made in the presence of various pH (A) at pH 2.0 (black), pH 3.0 (red), pH 4.0 (yellow), pH 6.0 (blue), pH 7.0 (purple), pH 8.0 (pink), pH 9.0 (green), pH 10.0 (gray), pH 11.0 (coral) and pH 12.0 (light green) at room temperature, and different temperatures (B) at 10°C (black), 20°C (gray), 30°C (yellow), 40°C (green), 50°C (blue), 60°C (purple) and 70°C (red) at pH 7.5, respectively. Values represent the mean ± SD of three analyses. The concentration of the Rv0045c protein was fixed at 0.35 mg/mL (20 mM Tris, pH 7.5).

To gain insight into the secondary structural elements in the Rv0045c protein, a circular dichroism (CD) spectroscopy was collected in the wavelength range from 240 to 190 nm at room temperature (25°C) and the pH range 2.0–12.0 (with an interval of 1.0, except pH 5.0 because the protein precipitates and may be too close to its pI).. The curves converged together in the range pH 6.0–10.0, but were nevertheless distorted and disordered at extreme pH (≤pH 4.0 and ≥pH 11.0). Near physiological conditions (at pH 7.0 and pH 8.0), the protein was much more stable and the negative trough at 216 nm with crossover at 195 nm is the characteristic feature of β-sheet secondary structure. The native state of the protein was estimated to entail 11∼14% α-helix, 54∼60% β-sheet, 4∼8% turn and 24∼26% random region, measured according to Yang and colleagues [14]. The high β-sheet content suggested that the Rv0045c protein possessed abundant β-sheet secondary structures, which is in accordance with the α/β-hydrolase fold [15] and implied that the Rv0045c protein may fall into the description of the α/β-hydrolase fold by Nardini and colleagues [16]. In the ranges pH 2.0–4.0 and pH 11.0–12.0, the structure of the protein had been denatured, showing that the conformations were quite different from those at pH 7.0 (as shown in Fig. 4A).

In order to assess the thermal stability of the protein, CD spectra was also collected at various temperatures (ranging from 10°C to 70°C, with an interval of 10°C) with the pH fixed at 7.5. The conformation of the Rv0045c protein was stable at temperatures ≤40°C and the curves converged together. The proportions of α-helix and β-sheet secondary structures at 30°C and 40°C (at 30°C: α  = 10.0%, β  = 61.3%, turn  = 4.0%; at 40°C: α  = 11.0%, β  = 58.1%, turn  = 7.6%) were similar to those for pH 7.0 and pH 8.0 at room temperature (25°C). When the temperature went down to ≤20°C, folding of the protein is consistent with inactivity (data not shown), although the percentages of α-helix and turn (at 20°C: α  = 16.1%, turn  = 14.8%; at 10°C: α  = 20.5%, turn  = 21.1%) notably increased. It was reported that the active site was fully available for substrate binding only when the protein was in the active and open conformation [16], and hence the Rv0045c protein adopts an inactive closed conformation at low temperatures, causing the enzyme activity to be extremely low. In contrast, when the temperature was increased to ≥50°C, the α-helical secondary structure was lost (e.g. α  = 4.7% at 60°C and α  = 4.8% at 70°C) and curves began to deviate from those for temeratures ≤40°C (as shown in Fig. 4B), which showed that the structure of the protein had been partially or largely denatured.

### Enzyme activity analysis of the Rv0045c protein

Based on the above results, and in order to test whether the Rv0045c protein has esterase activity, we experimentally analyzed the enzyme activity of the Rv0045c protein using *p*-nitrophenyl derivatives (*p*-nitrophenyl acetate (C_2_), butyrate (C_4_), caproate (C_6_), caprylate (C_8_), laurate (C_12_), myristate (C_14_) and palmitate (C_16_)) as substrates according to previously described methods [11], [17], [18]. As shown in Table 1, at pH 7.0 and 37°C, the Rv0045c protein could hydrolyze a wide range of *p*-nitrophenyl derivative (C_2_–C_14_) substrates, of which *p*-nitrophenyl caproate (C_6_) was effectively hydrolyzed. The substrates *p*-nitrophenyl acetate (C_2_) and *p*-nitrophenyl myristate (C_14_) were also visibly hydrolyzed with more than 50% maximal activity. In contrast, no enzyme activity towards longer *p*-nitrophenyl esters (C_16_) was detected (Table 1).

**Table 1:** Relative enzyme activity of the Rv0045c protein toward *p*-nitrophenyl derivatives at pH 7.0 and 37°C.

Substrate	Relative activity (%)^a^
*p*-nitrophenyl acetate (C_2_)	62.3
*p*-nitrophenyl butyrate (C_4_)	10.0
*p*-nitrophenyl caproate (C_6_)	100.0
*p*-nitrophenyl caprylate (C_8_)	14.2
*p*-nitrophenyl laurate (C_12_)	12.1
*p*-nitrophenyl myristate (C_14_)	77.9
*p*-nitrophenyl palmitate (C_16_)	ND

*ND*: Not detectable

a The specific activity toward *p*-nitrophenyl caproate (C_6_) corresponding to 3.5 U/mg protein/min was defined as 100%. And one hydrolase unit is the quantity of enzyme required to increase absorbance by 0.01 units at 405 nm per min.

**Figure 5:**
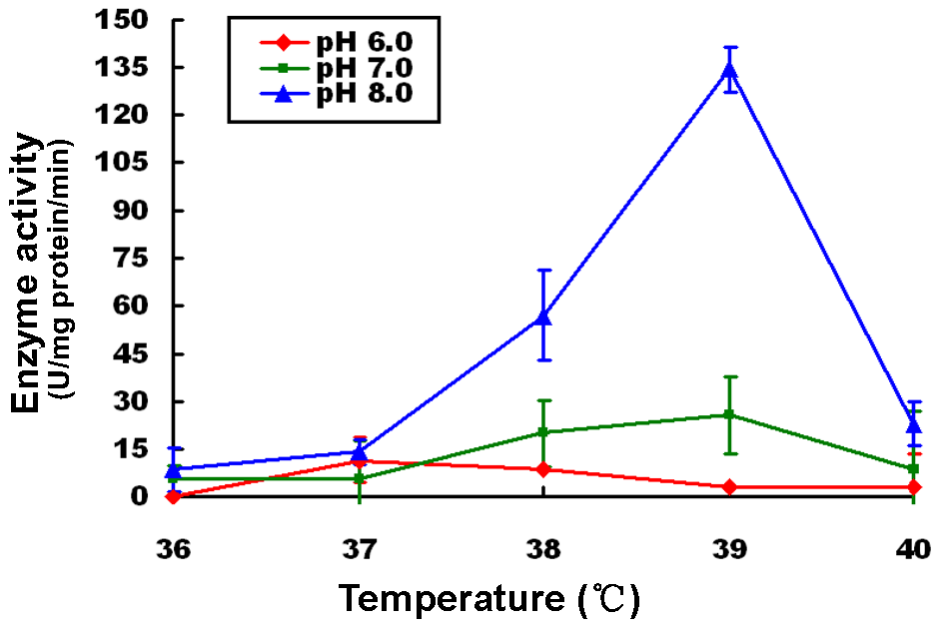
Effects of temperature and pH on enzyme activity of the Rv0045c protein. The enzyme activities were measured using *p-butyrate caprylate (C_6_)* as substrate in the presence of mild temperatures (36°C–40°C) at pH 6.0 (red), pH 7.0 (green) and pH 8.0 (blue). Values represent the mean ± SD of five analyses. The concentration of the Rvoo45c protein was fixed at 0.2 mg/mL (20 mM Tris, pH 7.5). The enzyme activities were expressed as units hydrolase/mg protein/min (one hydrolase unit is the quantity of enzyme required to increase absorbance by 0.01 units at 405 nm per min).

*M. tuberculosis* is known to presents a certain degree of resistance to aberrant potential of hydrogen. Activity of the Rv0045c protein was examined over a broad pH range from pH 2.0 to pH 12.0. No or poor activity was detected at ≤pH 4.0 and ≥11.0 (data not shown). Based on the CD spectroscopy data, the enzyme displays a conformation-dependent esterase activity, with activity declining dramatically or almost lost at ≤ pH 4.0 and ≥11.0 as a result of the enzyme becoming denatured. Activity was also too low to be detected at pH 9.0 and pH 10.0, for the reason that substrates spontaneously decomposed causing a deep background (data not shown). To determine the dynamic activity of the enzyme, we tested the activity using *p*-nitrophenyl caprylate (C_6_) as substrate at certain pH conditions (pH 6.0–8.0) in the temperature range around body temperature (from 36°C to 40°C), respectively. As shown in Fig. 5, the highest enzyme activity at pH 6.0 occurred at 37°C. At both pH 7.0 and pH 8.0, however, the optimal temperature for the enzyme activity is shown to be 39°C, In addition, the activity as a whole and also the highest activity at the optimal temperature exhibited a rapid and dramatic increase along with pH, suggesting that the Rv0045c protein adopted a pH-dependent activity in the pH range from 6.0 to 8.0, and can be described by the electrostatic potential distribution on the enzyme surface at alkaline pH making the substrate-binding and/or hydrolysis more effective [19].

## Discussion

Esterases or lipases are types of hydrolases which are widely distributed from prokaryotes to eukaryotes, and which are involved in lipid metabolism. As previously reported, *M. tuberculosis* is understood to contain more than 250 enzymes related to ester/lipid metabolism [6], [7]. In this study, we confirmed that the *M. tuberculosis* Rv0045c protein is a novel esterase. Compared with other esterases in the α/β-hydrolase fold family, two esterases Rv3487c [20] and Rv1399c [21] from the *M. tuberculosis*, both of which have been functionally characterized as esterases, shared no obvious sequence identity to our Rv0045c protein, in a multiple sequence alignment calculated using ClustalW software (data not shown).

All esterases in the α/β-hydrolase fold family have a nucleophile-histidine-acid catalytic triad evolved to efficiently operate on substrates with diverse chemical compositions or physicochemical properties [22], [23], [24]. Alignment among amino acid sequences showed that the active site G-X-S-X-G sequence motif within esterases is highly conserved (data not shown), and that the main catalytic residues (Ser89, Asp113, Ser206, His234) in the esterase ybfF [25] are also well conserved in our Rv0045c protein sequence. However, the Rv0045c protein shares as low as 23% amino acid sequence identity with ybfF. Additionally, residues around the active site in ybfF are quite divergent from those in the Rv0045c protein, suggesting that the Rv0045c protein has distinct substrate specificity and catalytic properties that set it apart from other esterases. As with the proteins Rv3487c [20] and Rv1399c [21], the Rv0045c protein can efficiently catalyze short-chain synthetic substrates (C_2_–C_8_), but can also hydrolyze *p*-nitrophenyl myristate (C_14_) with more than 50% of the maximal relative activity (Table 1).

Being the causative agent of most cases of tuberculosis, *M. tuberculosis* infects the lungs of the mammalian respiratory system and can persist in the human body at normal temperature (36°C–37°C) and pH (pH 7.3–pH 7.4) conditions for many decades. Thus, *p*-nitrophenyl acetate (C_6_) was used to determine the dynamic activity of the enzyme at mild pH conditions (pH 6.0–8.0) over the temperature range from 36°C to 40°C, which was around body temperature. Compared with the optimum reaction temperature of 30°C for Vlip509 [26], a new esterase from a strict marine bacterium, *Vibrio* sp. GMD509, the optimal temperature for the Rv0045c protein activity turned out to be 37°C at pH 6.0 and 39°C at both pH 7.0 and pH 8.0 (Fig. 5). This is probably the result of *M. tuberculosis* commonly living in the bodies of humans or animals whereas the *Vibrio* sp. GMD509 marine bacterium parasitizes in the eggs of the sea hare, a cold-blooded animal living at relatively low temperatures. It has also been observed that the average and the highest activity of the enzyme increased rapidly and dramatically following increased pH (Fig. 5), indicating that the metabolism of esters/lipids in this pathogen was more active when the circumstances become less favorable, especially more basic, and further suggests that *M. tuberculosis* becomes more pathogenic at alkaline pH.

*M. tuberculosis* is pathogenic to humans, and to some extent shows resistance to aberrant hydrogen potential. In our research, enzyme activities were determined over a broad pH spectrum (pH 2.0–12.0), yet little or no activity was detectable at extreme hydrogen potential (≤ pH 4.0 and ≥pH 11.0, data not shown). Results from CD spectroscopy analysis also indicated that, at extreme hydrogen potential (≤ pH 4.0 and ≥pH 11.0), the enzyme is partially or almost completely denatured, especially the α-helical secondary structure. These data suggest that the enzyme activity of the Rv0045c protein is conformation-dependent. Data from CD spectroscopy analysis showed that the Rv0045c protein is rich in β-sheet secondary structure, indicating that the enzyme should possess a very stable and substantial β-sheet core which helps to stabilize the architecture of the enzyme, thus ensuring that the pathogen can survive in strong environments. However, at extreme hydrogen potential (≤ pH 4.0 and ≥pH 11.0), the α-helical secondary structure of the enzyme was mostly denatured, and simultaneously the activity of the enzyme was not detectable. Based on the above evidence, it can be deduced that the β-sheet comprises the skeleton and backbone of the enzyme, while the α-helices or other secondary structure elements, e.g. turns, are required for the catalytic reaction. In addition, the conformation of the enzyme is very stable at temperatures ≤ 40°C, and the thermal denaturing temperature of the Rv0045c protein was determined to be 50°C, which can be utilized for dry heat sterilization to deactivate the enzyme and possibly even the pathogen.

The Rv0045c protein is just one of hydrolases involved in ester/lipid metabolism in *M. tuberculosis*, of which many members haven't been identified or haven't been studied. Biochemical characterization and functional analysis of those undefined esterase/lipase members should help to reveal the mechanism of ester/lipid metabolism of *M. tuberculosis*. In order to illustrate the relationship between the tertiary structure and function of the Rv0045c esterase, and to explain the molecular mechanism and principles of the Rv0045c protein participating in hydrolyzing esters, crystallography of the protein is under progress.

## Materials and Methods

### Protein expression

Based on the template of the whole genome of *M. tuberculosis*, the *Rv0045c* gene was amplified using a standard PCR procedure with primers R1 (5′-CGCGGATCCCTATCTGACGACGAACTGACC-3′, contained a *BamH I* digestible site) and R2 (5′-TCCGCTCGAGTCAGCGTGTGTCGAGCACCCC-3′, attached a *Xho I* site), and subcloned into the *BamH I* and *Xho I* sites of the pET28a vector (Novagen) with *6*×*his-tag* gene on N-terminal. The Rv0045c protein was overexpressed in *E. coli* BL21 (DE3) strain (Novagen) as a fusion protein with the 6×His-tag. Briefly, *E. coli* BL21 (DE3) carrying the *Rv0045c* gene was grown in LB medium at 37°C with 50 µg/mL kanamycin until the OD_600_ reached 0.6–0.8, and then induced with 0.3 mM IPTG at 16°C for 20 hrs. Protein expression was verified by SDS-PAGE analysis.

### Protein purification

For 1L culture, the cells harvested by centrifugation were homogenized in 80 mL buffer A (20 mM Tris, 150 mM NaCl, 10 mM Imidazole, pH 7.5) and disrupted by ultrasonication (400 W, 3 s/3 s, 4°C). Cell debris was removed by centrifugation at 15,000 rpm for 30 min at 4°C. The supernatant collected was loaded onto Ni Sepharose^TM^ 6 Fast Flow resin (GE Healthcare), which was pre-equilibrated with buffer A. The resin was washed with buffer B (20 mM Tris, 150 mM NaCl, 20 mM Imidazole, pH 7.5), and the objective protein was eluted with buffer C (20 mM Tris, 150 mM NaCl, 200 mM Imidazole, pH 7.5) and buffer D (20 mM Tris, 150 mM NaCl, 500 mM Imidazole, pH 7.5), sequentially. Collections were verified by SDS-PAGE analysis.

The target protein was dialyzed against buffer E (20 mM Tris, pH 7.5) at 4°C to remove the imidazole and salt, and then concentrated using a 10 kDa Centricon concentrator (Millipore). Concentrated protein was successively applied to Resource Q and Resource S 1 mL columns (GE Healthcare), and the protein was eluted from the column using buffer E with a gradient of NaCl from 0 M to 2 M. Finally, the protein was loaded onto a Superdex 75 10/300 GL column (GE Healthcare) in buffer F (10 mM Tris, 150 mM NaCl, 2 mM DTT, pH 7.5). All peak fractions were collected, and the protein purity was analyzed by SDS-PAGE.

### Mass spectrometry analysis

The gel strip was removed from the SDS-PAGE gel, cut into small pieces, washed with 100 µL 25 mM ammonium bicarbonate (pH 8.0) containing 50% acetonitrile for 15 min twice with vortexing. Gel pieces were dehydrated with 100 µL acetonitrile and completely dried with a Speed-Vac before tryptic digestion. The volume of the dried gel was evaluated and three volumes of 12.5 ng/mL trypsin (Promega) in 25 mM NH_4_HCO_3_ (freshly diluted) were added. The digestion was performed at 30°C overnight, and then the mixture was sonicated for 10 min and centrifuged. The supernatant was removed for matrix-assisted laser desorption/ionization time-of flight mass spectrometry (MALDI-TOF MS) analysis.

For MALDI-TOF MS analysis, 1 µL of the digested sample was spotted onto the MALDI target plate, and coated with 1 µL of matrix solution (5 mg/mL α-cyano-4-hydroxycinnamic-acid in 50% (v/v) acetonitrile and 0.1% (w/v) trifluoroacetic acid), then left to air-dry. Mass data were analysed with a prOTOFTM 2000 mass spectrometer interfaced with TOFWorksTM software (PerkinElmer/SCIEX). In this study, a 2-point external calibration of the prOTOF instrument was performed before acquiring the spectra from samples.

Protein identification was performed by searching for bacteria in the NCBI non-redundant database using the Mascot search engine (Matrix Science), using the following parameters: monoisotopic; mass accuracy, 0.1 Da; missed cleavages, 1.

### Circular dichroism spectroscopy analysis

During circular dichroism (CD) spectroscopy analysis, purified 6×His N-terminally tagged Rv0045c protein (0.35 mg/mL) was solubilized in 20 mM Tris (pH 7.5) and measured in the presence of room temperature with different pH (pH 2.0–pH 12.0) and pH 7.5 with different temperatures (10°C–70°C), respectively. UV CD spectra between 190 and 250 nm were collected on a JASCO 715 spectropolarimeter (JASCO) using 1 mm quartz cuvettes containing 200 µL of the protein solutions, with a data pitch of 0.1 nm, bandwidth of 2.0 nm and scanning speed of 50 nm/min. Every sample was measured in triplicate, and data were analyzed using the Jasco Jwsse 32 secondary structure estimation software.

### Enzyme activity analysis

Enzyme activity of the Rv0045c protein was measured as previously described [11], [17], [18] using seven substrates: *p*-nitrophenyl acetate (C_2_), butyrate (C_4_), caprylate (C_6_), caproate (C_8_), myristate (C_12_), laurate (C_14_) and palmitate (C_16_). The activities were determined by applying 10 mM *p*-nitrophenyl esters (C_2_–C_16_) as substrates at different pH (pH 6.0–pH 9.0) and different temperature (36°C–40°C). The substrate of *p*-nitrophenyl caprylate (C_6_) was also used to estimate the dynamic activity of the enzyme at pH from 6.0 to 8.0 in the presence of mild temperatures (36°C–40°C). .For each standard assay, 50 µL 10 mM sodium taurocholate, 20 µL 10 mM substrate (dissolved in chloroform), and 420 µL Britton-Robinson buffer solution with different pH (pH 6.0–pH 9.0) were mixed in 1.5 mL Eppendorf tube separately, and then 10 uL protein (0.2 mg/mL) was added into each tube. After incubating at different temperatures for 15 min, the reaction was terminated by adding 700 µL 5∶2 (v/v) acetone/hexane solution. The mixture was then centrifuged at 4,600 g for 2.5 min at room temperature and the OD_405_ of the lower phase was measured. Simultaneously, three controls were made: one prepared by adding the Rv0045c protein after adding acetone/hexane solution to observe instant hydrolysis; another prepared by substituting substrate solution with chloroform; and the other prepared by substituting the Rv0045c protein with 20 mM Tris (pH 7.5). Five parallel tests were repeated for every substrate at different pH and temperatures.
